# Clinical performance of narrow-diameter titanium–zirconium implants in immediately loaded fixed full-arch prostheses: a 2-year clinical study

**DOI:** 10.1186/s40729-021-00312-3

**Published:** 2021-04-16

**Authors:** Fatih Mehmet Coskunses, Önjen Tak

**Affiliations:** 1 Department of Oral and Maxillofacial Surgery, Faculty of Dentistry, University of Kocaeli, 41190 Kocaeli, Turkey; 2 Department of Prosthodontics, Faculty of Dentistry, Istanbul Okan University, Tuzla, Istanbul, Turkey

**Keywords:** Narrow-diameter implants, Immediate loading, Fixed full-arch prostheses, Full edentulism

## Abstract

**Objectives:**

The aim of this study was to evaluate the outcomes of immediate fixed full-arch prostheses supported by axial or tilted narrow-diameter Ti-Zr implants (3.3 mm) (Roxolid®, Institut Straumann® AG, Basel, Switzerland) (NDIs) in combination with standard-diameter implants up to 2 years’ follow-up.

**Materials and methods:**

The study was conducted at Kocaeli University Faculty of Dentistry from 2016 to 2018. 37 jaws of 28 patients with an average age of 52 years were rehabilitated with fixed full-arch prostheses supported by 179 implants. Cumulative survival rate (CSR), implant success, marginal bone loss (MBL), and prosthetic survival rate as well as complications were analyzed.

**Results:**

Total CSR of 99.4% and 98.5% for all and narrow implants respectively have been observed at 2 years’ follow-up. No prosthesis failures were observed, yielding a cumulative prosthetic survival rate of 100%. The NDIs achieved 0.63 mm MBL at 1 year and 1.02 mm at 2 years. The mean MBL at 1 year was 0.51 mm (mandible 0.63 mm/maxilla 0.41 mm) and 0.73 mm (mandible 0.90 mm/maxilla 0.43 mm) at 2 years. Both implant angulation and loading protocol did not influence the MBL.

**Conclusions:**

The combination of narrow-diameter implants with standard-diameter implants in immediate fixed full-arch rehabilitation has a good prognosis to become a new standard of care for severely atrophic jaws.

**Clinical relevance:**

The use of narrow-diameter implants in fixed full-arch rehabilitations in atrophic ridges would be a successful and predictable treatment approach.

## Introduction

Edentulism is a globally common problem associated with physical, emotional, social, and psychological wellbeing and self-esteem of the patient [1, 2]. The most important negative consequences of edentulism are decreased chewing efficiency, phonation problems, unsatisfied esthetic appearance, decreased self-confidence, and overall reduced oral health–related quality of life of the patient [1, 2].

The rehabilitation of edentulous jaws may be complex due to reduced bone volume with long-term edentulism [3]. Bone augmentation is often associated with higher surgical risks of morbidity and complications, higher financial costs, and longer time to complete the treatment [3–5]. In order to overcome limitations, different therapeutic alternatives, such as distal cantilever, short implants [6, 7], implants placed in the pterygoid region, the tuber or the zygoma [3, 8, 9], or tilted implants [1–5, 10, 11] have been proposed. Another satisfactory solution in atrophic cases is narrow-diameter implants (NDIs), which reduce the need for augmentation procedures.

Titanium–zirconium alloy (Ti-Zr; Roxolid; Institut Straumann AG, Basel, Switzerland) has been developed as a new implant material with increased biomechanical properties and excellent biocompatibility that enable the use of NDIs even in clinically challenging situations. The most vital benefits of NDIs for the patient are the reduction in complexity, duration, and costs of treatment due to the less frequent requirement for bone grafting [12].

Immediate loading protocol of the dental implants is widely reported in recent reviews, systematic reviews, and metal-analyses [4, 13, 14]. Gallucci et al. stated that the existing literature provides high evidence that immediate loading of microtextured dental implants with one-piece fixed interim prostheses in both the edentulous mandible and maxilla is as predictable as early and conventional loading [15].

The aim of this study was to evaluate the prognosis of fixed full-arch prostheses supported by NDIs (3.3 mm in diameter Ti-Zr implants) with a combination of standard-diameter implants and to compare the survival and success rates and marginal bone level (MBL) changes of Ti-Zr implants up to 2 years of function.

## Materials and methods

This study reports the clinical outcomes of an immediately loaded implant-supported fixed full-arch prosthesis in the treatment of patients between 2016 and 2018. The study was conducted in accordance with the Declaration of Helsinki, and with the written informed consent of the patients. The study was approved by the Kocaeli University ethics committee (Authorization Number = KU GOKAEK 2018/209)

### Patient selection

The inclusion criteria of the study were either edentulous jaws or jaws with teeth with a poor long-term prognosis that are planned for extraction. Patients of any gender who were at least 18 years old and medically able to undergo implant surgery and restorative procedures and had acceptable oral hygiene were included in the study. Patients who had unstable systemic diseases, were undergoing radiotherapy or chemotherapy, and had severe bruxism or another contraindication of implant therapy were not treated. Cases with suspected poor motivation to return for follow-up visits were not treated. Smoking was not an exclusion criterion; however, all smokers were informed about the failure risk and prompted to stop smoking successfully. Patients with periodontal diseases had either treatment of condition before surgery or extraction of teeth at least 3 weeks prior to implant surgery. All consecutive patients meeting these criteria were included in the study.

The study included 37 jaws (19 mandible/18 maxilla) of 28 patients, 11 females and 17 males, with an average age of 52 years (range, 23–72 years). Each patient has been treated with an implant-supported screw-retained fixed full-arch prosthesis supported by a minimum of four implants in each jaw. A total of 179 Straumann Roxolid Bone Level Tapered SLA implants (Institut Straumann AG, Basel, Switzerland) with various lengths (minimum 10 mm) were inserted. Sixty-seven of implants (39 in the mandible and 28 in the maxilla) (length 12 mm: 33n, 14 mm: 25n, 16 mm: 8n, 10 mm: 1n) were in narrow diameter (3.3mm, 38%) while 15 implants were 4.8 mm in diameter (8%). The remaining 97 implants were 4.1 mm in diameter (54%). Surgery (FMC) and prosthetic procedures (OT) were performed by the authors.

Twelve patients (42%) had a systemic condition: cardiovascular condition (*n* = 10 patients), thyroid (*n* = 1 patient), diabetes (*n* = 2 patients). One patient presented more than one condition. Six patients (21%) were smokers before treatment.

The conditions of the opposing arch included the following distributions: natural teeth (*n* = 2), tooth-supported fixed dental prosthesis (FDP) (*n* = 4), implant-supported FDP (*n* = 1), implant and tooth supported FDP (*n* = 12), full-arch implant-supported prosthesis (*n* = 9). None of the opposing jaws were restored with removable prosthetic solutions.

### Presurgical preparation

**Fig. 1:**
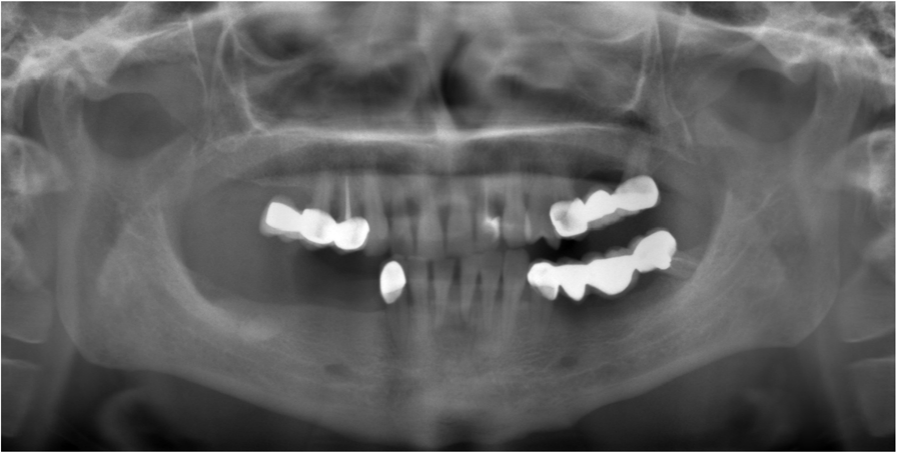
Panoramic X-ray evaluation of patient

**Fig. 2:**
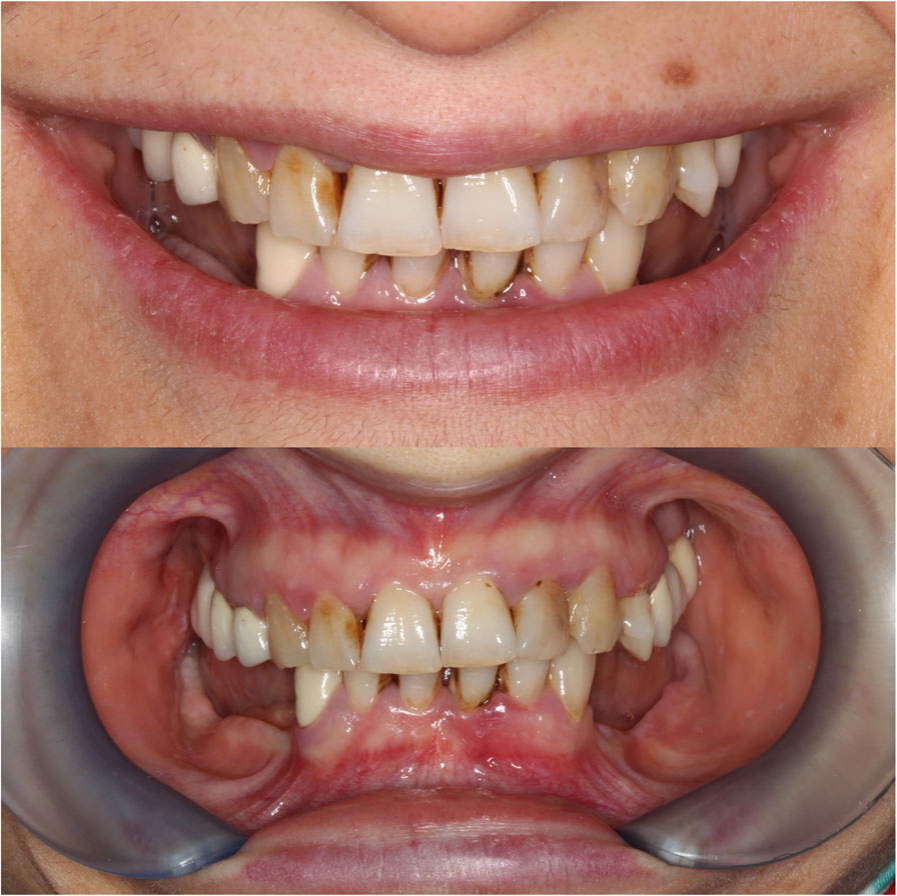
Clinical view of patient 1 in diagnosis and treatment planning stage

All patients were reviewed for medical histories, together with clinical observation and radiographic exams with an orthopantomography and cone beam computed tomography (CBCT) scan for diagnosis and treatment planning (Figs. 1 and 2). Analysis of the CBCT scans was used to select the optimal implant diameter and length, as well as the location for immediate function. The most distal implant was planned at the first molar region if possible with a minimum of 10 mm. If this was not possible, angulation of distal implants (17–30°) was performed in order to achieve a longer A-P spread of implants and minimize cantilever.

Evaluation of the patients’ esthetics and function intra- and extra-orally as well as on diagnosis models was essential to identify the patient’s smile line and vertical occlusal space. A diagnostic wax try-in denture was created in order to determine the relation between the teeth and the alveolar ridge. Phonetics, facial support, and esthetic parameters also sometimes led us to bone reduction for prosthetic aims. A surgical guide (a duplicate of the provisional full-arch prosthesis) was fabricated from transparent heat-processed acrylic resin to orient the surgery. Centric occlusion of the patient was taken with silicone impression intra-orally or on the diagnostic cast models mounted in the articulator. By this way the most proper bucco/palato lingual alignment of implants and the proper vertical dimension and centric relation could be maintained during the whole provisionalization procedure.

### Surgery

Surgical procedures were performed under local anesthesia (4% articaine with 1:100,000 epinephrine; Ultracain, Sanofi-Aventis; PharmaVision, Istanbul, Turkey). Antibiotics (amoxicillin 875 mg + clavulanic acid 125 mg; Amoklavin, Deva ilac, Istanbul, Turkey) were given 1 h prior to surgery and daily for 5 days thereafter. Corticosteroids (methylprednisolone 1mg/kg; Prednol, Mustafa Nevzat ilac, Istanbul, Turkey) were given daily using a tapering dose regimen (1mg/kg to 16 mg) from the day of surgery until 4 days postoperatively. Anti-inflammatory drugs (25 mg deksketoprofen, Arveles, Ufsa Ilac, Istanbul, Turkey) were also given after surgery.

The crestal incision was placed lingually and the full-thickness mucoperiostal flap was raised. In cases which need bone reduction, vertical incisions were placed distal to the first molar area. For the dentate patients, before extracting the teeth, the vertical dimension of the patient was determined by the measurement of marked points between the chin and the nose. The remaining teeth were extracted and debrided atraumatically. Planned bone reduction and implant bed preparation was done with a surgical saw and burs parallel to the occlusal plane. A fabricated surgical guide and the Straumann Pro Arch Guide (Institut Straumann AG, Basel, Switzerland) aided for the position and angulation of implants. Insertion torque was confirmed with a torque wrench (Institut Straumann AG, Basel, Switzerland) and Osstell ISQ device (Osstell, Sweden). Implants that achieved primary stability (insertion torque) of at least 35 Ncm and RFA value of at least 65 were immediately loaded. In the case 2 of the implants did not achieve the immediate loading criteria in a 4-implant scenario, conventional loading protocol was applied. In the cases with 6 or 8 implants, when 4 of the implants meet the loading criteria, a provisional prosthesis was delivered on eligible implants.

**Fig 3:**
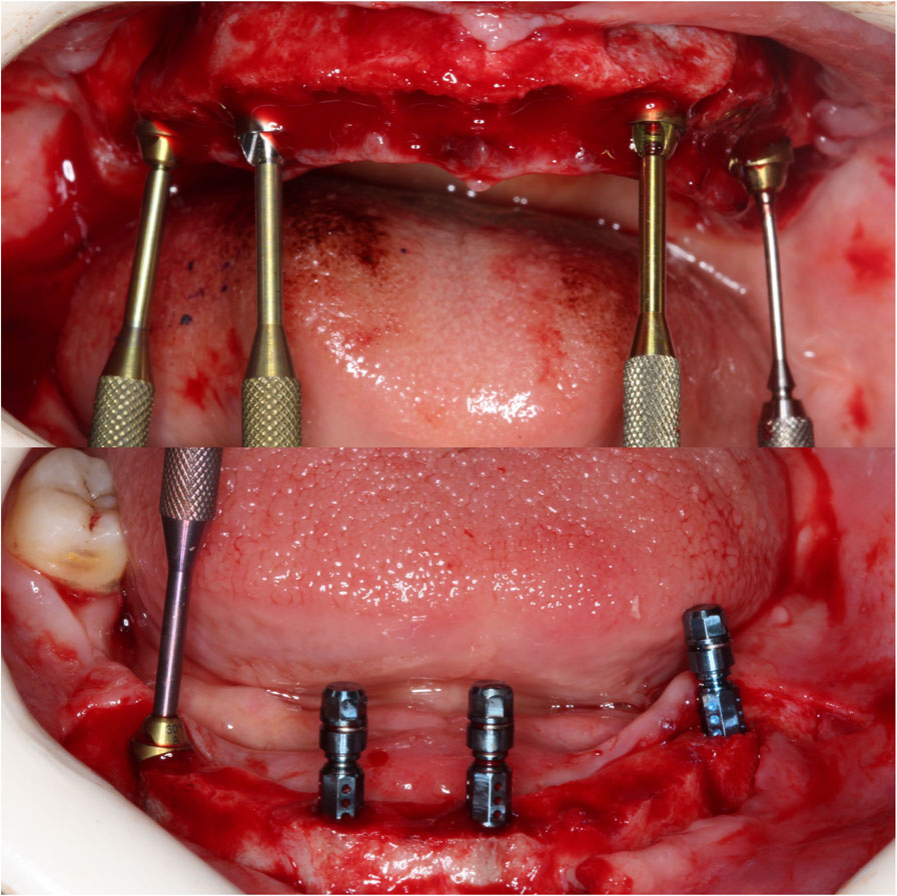
Insertion of implants, placement of SRA abutments in surgery

**Fig. 4:**
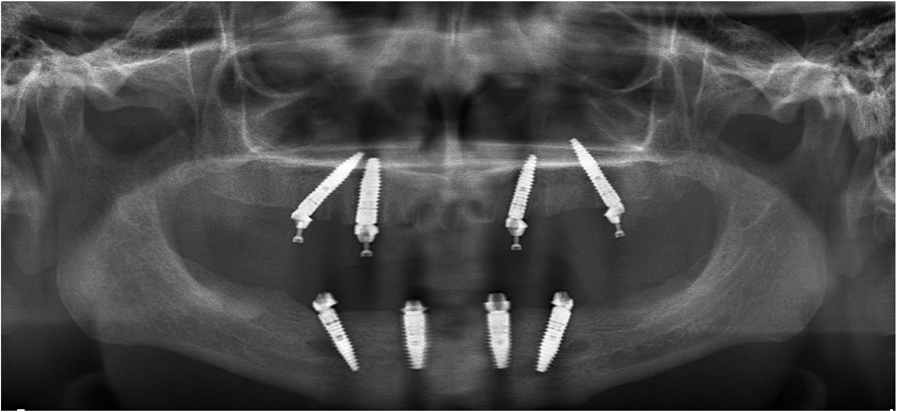
Panoramic X-ray after surgery

Once implants were placed, screw-retained abutments (SRA, Institut Straumann AG, Basel, Switzerland) were placed onto the implants and torqued (Figs. 3 and 4).

### Immediate fixed provisional full-arch prosthesis

**Fig. 5:**
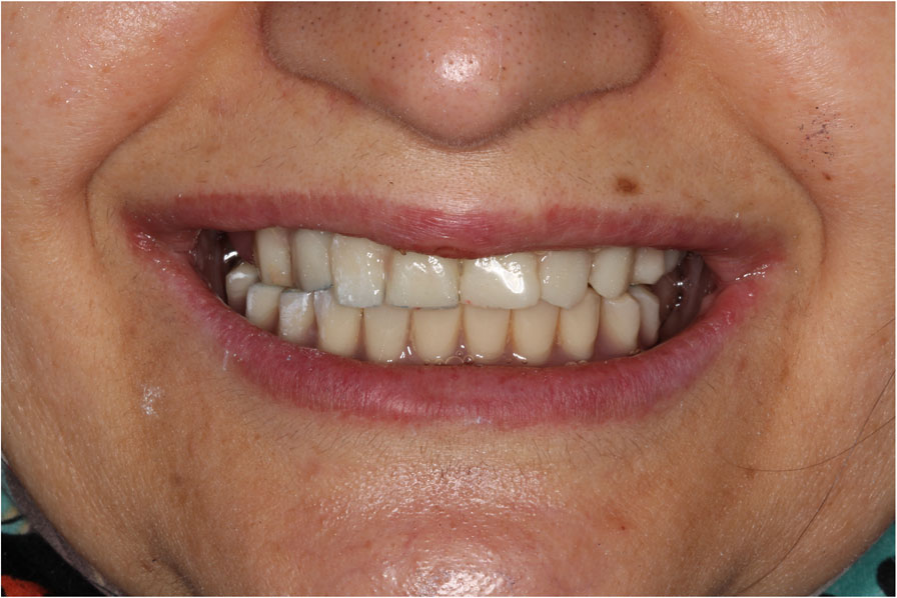
Provisional prosthesis in the same day with surgery

A silicone impression material was used to line the intaglio surface of the fabricated denture to identify the position of abutment access holes. After trimming of the holes, the non-engaging titanium copings (Institut Straumann AG, Basel, Switzerland) were screwed, and the denture was placed intra-orally to check passivity and fit of the denture around the copings. Following the verification of the proper seating and alignment of the denture with pre-operative bite registration, the titanium copings were fixed to the denture using a quick-setting, self-curing denture repair resin based on diacrylate (Qu resin, Bredent GmbH & Co.KG; Senden, Germany) intra-orally. After the polymerization, the denture was unscrewed and the access and the passivity of it were verified in the patient’s mouth. The height of the titanium copings was reduced, the buccal and lingual flange extensions were trimmed, and the intaglio surface of the fixed denture was shaped convex and polished well to maintain oral hygiene. Cantilever stresses were minimized by reducing the distal cantilever length of the lower and upper arches with maximum of 10 teeth. The prosthetic screws were torqued to 15 Ncm. The occlusion was adjusted, centric and lateral contacts were limited to the intercanine zone (Fig. 5).

### Definitive (hybrid) prosthesis

A definitive prosthesis was delivered 4 to 6 months later. Open tray impression posts (Institut Straumann AG, Basel, Switzerland) were placed and firmly seated and luted together using a light cure material or pattern resin. The impressions were taken using polyether impression material (Impregum, 3M ESPE, Seefeld, Germany). Using a verification jig was a key factor to verify the accuracy of the impression and master implant model before the fabrication of the substructure of the prosthesis.

**Fig. 6:**
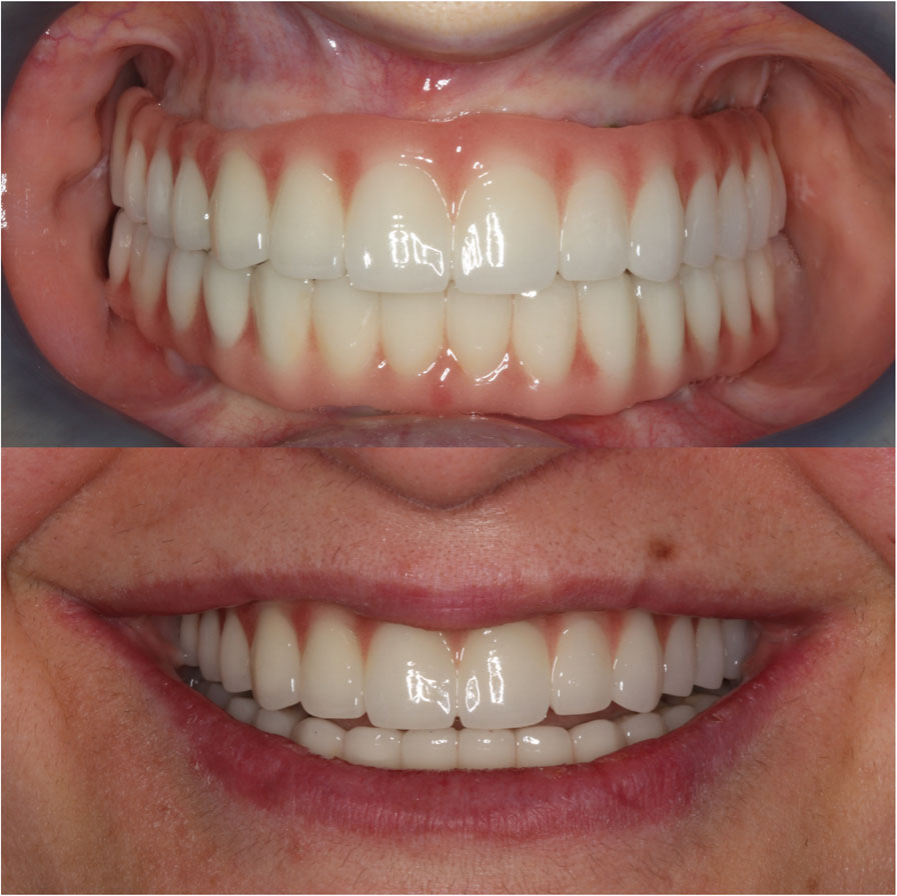
Definitive prosthesis of patient

The substructure (framework) was designed and fabricated using titanium or cobalt–chrome alloy. The try-in of the substructure was evaluated visually and with an X-ray to confirm that each abutment was seated properly. The Sheffield test was also used to verify the passive fit of the framework with each abutment. In the esthetic try-in appointment, phonetics, esthetics, smile line, and lip support were checked as a typical denture. The design of the tissue interface of the hybrid prosthesis was convex, smooth, and highly polished to provide the tissue to roll over the prosthesis on the buccal and lingual aspects and that the patient can easily clean the intaglio surface of the prosthesis. After the occlusal, esthetic, phonetic, and functional adjustment of the definitive prosthesis, the prosthetic screws were torqued with a torque wrench to 15 Ncm. Screw access holes were sealed with a resin composite followed by blocking out screw access holes with Teflon tape. A night guard was always provided (Fig. 6).

### Follow-up

**Fig. 7:**
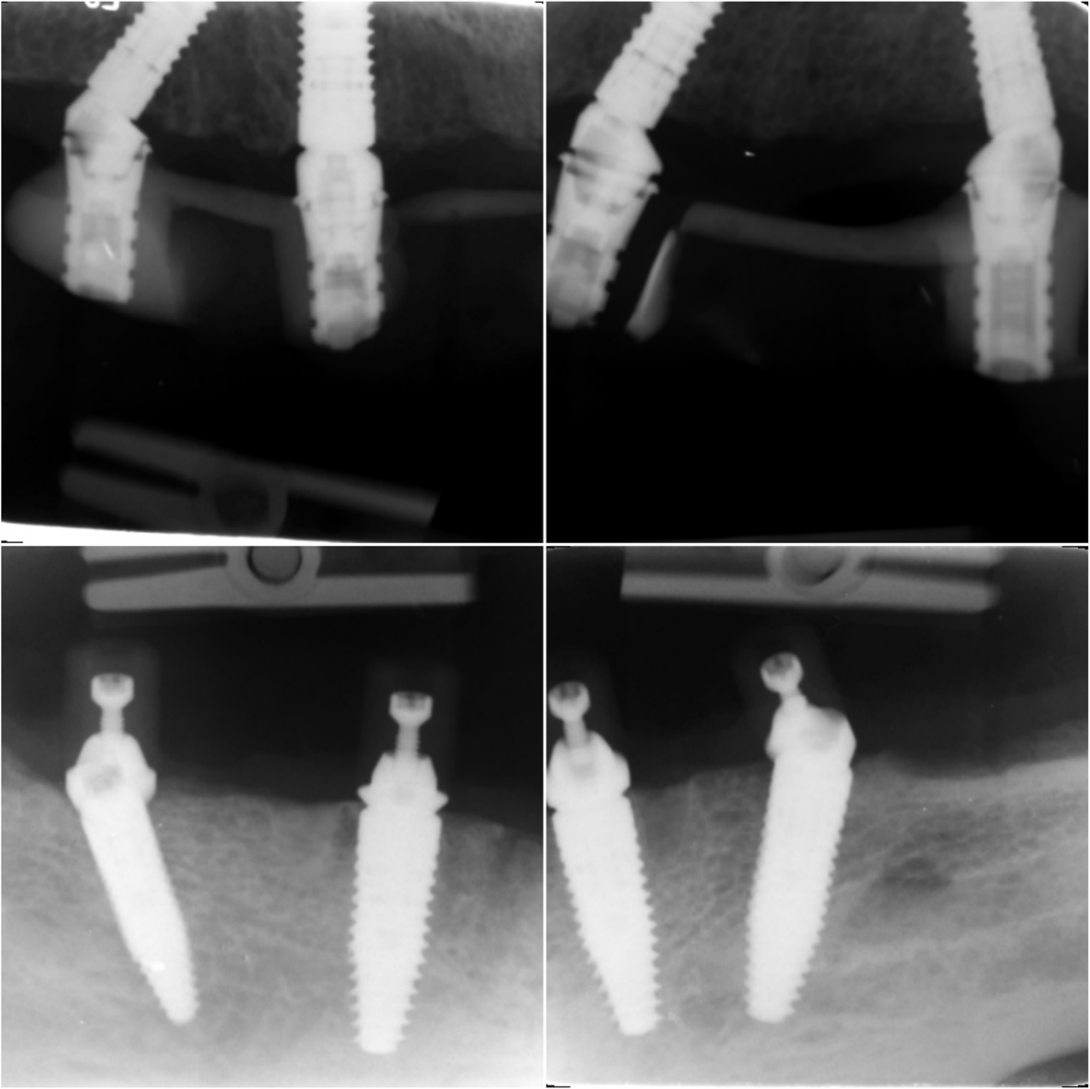
Radiological follow-up of patient was performed with paralleling X-ray

Follow-up examinations were performed at 7 days; 1, 3, and 6 months; 1 year; and thereafter every 6 months. The radiographic evaluation was done at 6 months and 1 and 2 years of follow-up. The paralleling technique was used for intraoral X-ray diagnostics with the holders that align the sensor at a right angle to the central beam and thus parallel to the implant axis. The analysis of marginal bone level was assessed with image analysis software (ImageJ version 1.51 for Mac, National Institutes of Health, USA). The marginal bone level was assessed on mesial and distal sides from most coronal bone to the implant neck (Fig. 7).

Implant success and survival was assessed according to the criteria accepted in the International Congress of Oral Implantologists Consensus Conference for Implant Success in Pisa, Italy, October 2007. Prosthesis success is considered as stable and functionally serving provisional and permanent prosthesis. Biological as well as mechanical or prosthetic complications were also recorded.

### Data analysis

Statistical analysis was performed using IBM SPSS Software Version 23. The primary parameter was to assess the cumulative survival rate (CSR) of implants and to perform a comparison between NDIs and others and also immediately loaded and late-loaded implants. Cumulative success rate of implants was also assessed. The other objective of the study was to evaluate MBL compared to baseline 1 and 2 years after implant placement on the following subgroups:

- implant diameter (3.3 mm), (4.1 mm) and (4.8 mm)
- tilted (30°) vs. axial implants
- time of implant loading (immediate, late)
- localization of the implant in the jaw (mandible, maxilla)
- the number of implants in the jaw (4, 6, or 8 implants)

Descriptive MBL statistics were computed for mean over distal and mesial bone loss measurements. Two subgroup means were compared by Student’s *t* test. Combined subgroups (e.g. angle and diameter) were compared by two-way ANOVA for each subgroup (main effects) and interaction between both subgroups. The test results were provided together with detailed test statistics, *p* values, and 95% confidence intervals (where appropriate). The STROBE checklist for the present paper was completed. The statistical analysis was reviewed by an independent statistician.

## Results

A total of 179 implants (Roxolid; Institut Straumann AG, Basel, Switzerland) (96 maxilla and 83 mandible) were placed in 28 patients (17 males and 11 females, mean age at surgery 52 years, range 23–72 years).

Thirty-seven jaws were restored with 19 mandibular and 18 maxillary prostheses. In 29 of the jaws (130 of 179 implants), provisional prostheses were delivered on the same day of surgery which met the objective criteria of immediate loading. Two patients with 3 jaws gave up immediate loading on the day of surgery. In one case, 2 of 4 implants did not achieve the 35-Ncm insertion torque, and in two cases with double jaws, 3 of 6 implants in the maxilla did not meet loading criteria and loading of both maxilla and mandible was delayed. Sixteen of 19 prostheses in the mandible were supported by 4 implants while 3 of them were supported by 6 implants. In the maxilla, 7 prostheses were supported by 4 implants and 10 were supported by 6 implants. One patient had 4 implants in the provisional and 8 implants in the permanent prosthesis period. Sixty-seven narrow-diameter Ti-Zr implants were inserted in 24 of 28 patients. Implant distribution according to diameter and loading protocol is detailed in Table 1. The follow-up period was 6–24 months (mean 15.8 ± 6.3 m).

**Table 1:** Implant distribution according to implant diameter and loading protocol

Loading	Maxilla			Mandible			Total
	3.3 mm	4.1 mm	4.8 mm	3.3 mm	4.1 mm	4.8 mm	
Late	5	15	2	10	14	3	49
Immediate	23	43	8	29	25	2	130
Total	28	58	10	39	39	5	179

### Cumulative survival and success rate

Overall, one implant was lost in the mandible with a diameter of 3.3 mm (length 14 mm) in the first 3 months. In this patient, a new implant with a diameter of 4.1 mm was placed with late loading protocol and the provisional prosthesis was modified and supported by three implants until definitive prosthesis loading. This resulted total cumulative survival rate (CSR) of 100% for 4.1 mm (*n*: 97) and 4.8 mm (*n*: 15) implants while 98.5% for NDIs which was statistically insignificant by means of diameter of implant and loading protocol after 2 years’ follow-up (*p* = 0.193/NDIs and others (4.1 and 4.8mm), (*p* = 0.227/NDIs and 4.1 mm), (*p* = 0.634/NDIs and 4.8mm), (*p* = 0.588/NDIs with immediate and late loading) (Table 2). Total CSR of all implants was 99.4% which was statistically insignificant by means of loading protocol (*p* = 0.538/all implants immediate and late loading) (Table 2). The survival rate of the prostheses was 100% after 2 years. Another 3 implants (2 in mandible and one in maxilla) in 3 patients presented biological complications that showed > 4-mm peri-implant pocket, > 2-mm MBL, and bleeding on probing. In all patients, the problem was solved through surgical treatment that aims to clean the implant surface mechanically, and disinfecting the surface with 0.2% chlorhexidine and laser. Implants maintained their healthy condition without affecting implant survival. No further biological complications were registered. The success rate of all implants was 98.3% in 2 years.

**Table 2:** Cumulative survival rates (CSR) for implants (total/narrow) inserted

Duration	All implants/NDIs	Failed	Withdrawn	CSR%		
				All^#^	NDIs* ^	Others (4.1 and 4.8 mm)
Placement–6 months	179/67	0/1	0/0	99.4	98.5	100
6 months–1 year	142/58	0/0	0/0	99.4	98.5	100
1–2 years	38/17	0/0	0/0	99.4	98.5	100

*NDIs to others; *p* = 0.193, NDIs to 4.1 mm; *p* = 0.227 and NDIs to 4.8 mm; *p* = 0.634 (Pearson’s chi-square)

^NDIs with immediate to late loading; *p* = 0.588 (Pearson’s chi-square)

^#^All implants with Immediate to late loading; *p* = 0.538 (Pearson’s chi-square)

### Marginal bone loss

The mean MBL at 1 year was 0.51 mm (*n* = 142) (mandible 0.63 mm/maxilla 0.41 mm/*p* = 0.009) and 0.73 mm (*n* = 38) (mandible 0.90 mm/maxilla 0.43 mm/*p* = 0.032) in the second year (Table 3).

**Table 3:** Marginal bone loss of implants

Bone loss (mm)	Maxilla	Mandible	Tilted	Axial	Total
1st year	0.41 ± 0.38	0.63 ± 0.60*	0.61 ± 0.63	0.45 ± 0.41	0.51 ± 0.51
2nd year	0.43 ± 0.32	0.90 ± 0.74^#^	0.90 ± 0.75	0.61 ± 0.57	0.73 ± 0.66

Statistical significance: *p = 0.009* and ^#^*p* = 0.032

The NDIs (3.3 mm) achieved 0.63 mm (*n* = 58) MBL at 1-year data and such result was not significantly different from 4.1 (0.46 mm (*n* = 69) and 4.8 (0.32 mm (*n* = 15) mm diameter implants (*p* = 0.05). The MBL of narrow-diameter implants was 1.02 mm (*n* = 17) while it was 0.44 mm (*n* = 16) for the 4.1-mm implant in the second year (Table 4). The difference between the 3.3- and 4.1-mm diameter was statistically significant in the second year (*p* = 0.035) (Table 4).

**Table 4:** Marginal bone loss of implants in different diameters

Bone loss (mm)	3.3 mm	4.1 mm	4.8 mm	*P* value
1st year	0.63 ± 0.44	0.46 ± 0.54	0.32 ± 0.49	0.05
2nd year	1.02 ± 0.74*	0.44 ± 0.32*	0.64 ± 0.85	0.035

*Statistical significance (*P* < 0.05) between 3.3- and 4.1-mm implants

The mean MBL at 1 year was 0.53 mm in immediately loaded implants (*n* = 110) and 0.46 mm in late-loaded implants (*n* = 32). In the second year, MBL in immediately loaded and late-loaded implants was 0.67 mm (*n* = 34) and 1.24 mm (*n* = 4) respectively. The difference at MBL was not significantly affected by loading protocol in the first (*p* = 0.522) and second years (*p* = 0.099) (Table 5).

**Table 5:** Marginal bone loss of implants in different loading protocols

Bone loss (mm)	Immediate	Late	*P* value
1st year	0.53 ± 0.53	0.46 ± 0.40	0.522
2nd year	0.67 ± 0.56	1.24 ± 1.23	0.099

The mean MBL of tilted and axial implants was 0.61 ± 0.63 mm and 0.45 ± 0.41 mm at 1 year, respectively. In the second year, MBL of tilted and axial implants was 0.90 ± 0.75 mm and 0.61 ± 0.57 mm, respectively. The MBL differences between tilted and axial implants were not statistically significant at 1 year (*p* = 0.072) and in the second year (*p* = 0.181) (Table 3).

The mean MBL of implants in which the rehabilitation was done with 4 implants was 0.63 mm (*n* = 80/Sd 0.59 mm) while it was 0.35 mm (*n* = 54/Sd 0.33 mm) in 6 implant-supported prostheses at 1 year. This result was statistically different at the 0.05 level (*p* = 0.0060). The significance could not be calculated for 2 years’ data because of inadequate sample number.

Implant localization (mandible), number of implants (smaller numbers) and diameter (narrow) were associated with greater bone loss in 1 (*p* = 0.002, *p* = 0.001 and *p* = 0.00 respectively) and 2 years. (*p* = 0.048, *p* = 0.01 and *p* = 0.008 respectively) These findings are supported by Spearman’s correlation and regression analysis.

### Prosthesis

Twenty-nine fixed provisional prostheses were incorporated on the same day after the surgery. All of these prostheses were all-acrylic prostheses without metal frameworks. The fracture of the screw-retained fixed provisional prosthesis was recorded in 4 patients (14,3%). The incidence of fracture of the provisional prostheses in the present study was 16.1% (5 prostheses) of the total cases. All the fractures had been repaired in the clinical setting without sending the prostheses back to the laboratory. To date, no fracture of the 37 definitive prostheses has been reported. Chipping of the ceramic occurred in 5.4% of the definitive prostheses in 2 patients. Tooth detachment of the provisional fixed acrylic prosthesis was recorded in two patients. The screw loosening occurred in a provisional prosthesis.

## Discussion

Atrophy of the jaws is the main guide in the decision process of immediate fixed restorations which dictates the number of implants, angulation, and diameter/length of the implant. According to the current literature, especially in the case of severely resorbed alveolar ridges, placement of a narrow or tilted implant is a suitable and feasible treatment alternative to avoid additional surgical invasive procedures [1, 5, 10, 11, 16]. In previous papers, the use of implants with narrow diameters of 3.3 to 3.5 mm and Ti-Zr alloy are well documented in all indications including load-bearing posterior regions with promising success rates [12, 17–19]. However, the data of NDIs used in the treatment of fixed full-arch prosthesis was inadequate in the literature.

Peri-implant bone level changes of implants supporting immediate fixed full-arch prosthesis were reported by some researchers. Crespi et al. reported mean 1.10 ± 0.45 mm MBL for axial maxillary implants (*n* = 48 implants) and 1.11 ± 0.32 mm MBL for tilted maxillary implants (*n* = 48 implants) at the 36-month evaluation [20]. In the mandible, the mean peri-implant MBL of 1.06 ± 0.41 mm for axial implants (*n* = 40) and 1.12 ± 0.35 mm for tilted implants (*n* = 40) was found at 36 months’ follow-up in the same study. In a study of Malo et al., the average peri-implant bone loss in the mandible was 1.7 mm ± 0.6 mm at 5 years while it was 1.6 ± 0.4 mm in the maxilla at 3 years [21].

Patzelt et al. evaluated 13 (487 initially identified) papers which met inclusion criteria in their systematic review. A number of 4804 implants was evaluated and the mean MBL (12 months) of maxilla, mandible and combined were 1.0 ± 0.5 mm, 0.8 ± 0.4 mm, and 0.9 ± 0.5 mm, respectively and the mean MBL (24 months) of the maxilla, mandible, and combined were 0.8 ± 0.4 mm, 1.0 ± 0.4 mm, and 0.9 ± 0.4 mm, respectively. The bone loss in axial implants (12 months) of the maxilla, mandible, and combined were 0.8 ± 0.3 mm, 0.9 ± 0.5 mm, and 0.8 ± 0.4 mm, respectively. The bone loss in axial implants (24 months) of maxilla, mandible and combined were 0.8 ± 0.4 mm, 1.0 ± 0.4 mm, and 0.9 ± 0.4 mm, respectively. The bone loss in tilted implants (12 months) of the maxilla, mandible, and combined were 0.7 ± 0.4 mm, 0.8 ± 0.5 mm, and 0.8 ± 0.4 mm, respectively. The bone loss in tilted implants (24 months) of the maxilla, mandible, and combined were 0.9 ± 0.4 mm, 0.9 ± 0.4 mm, and 0.9 ± 0.4 mm respectively. They reported no significant differences between maxillary versus mandibular arches and axially versus tilted implants [5]. Also, Menini et al. evaluated the outcomes of axial and tilted implants supporting fixed full-arch dentures for the immediate rehabilitation of edentulous maxilla, after at least 1 year of function in their meta-analysis. The MBL was obtained from 6 studies and the mean MBL was 0.75 mm (tilted, 0.77 mm; axial, 0.73 mm) which was not statistically significant [22].

In the present study, the mean MBL was 0.51 ± 0.51 mm and 0.73 ± 0.66 mm at 1 and 2 years, respectively. In the maxilla, MBL resulted in 0.41 ± 0.38 mm in 1 year and 0.43 ± 0.32 mm in the second year while it was 0.63 ± 0.60 mm and 0.90 ± 0.74 mm in the mandible respectively. The difference in mean MBL between mandible and maxilla was significant in one (*p* = 0.009) and 2 years’ (*p* = 0.032) follow-up. This result is not in accordance with the systematic review of Patzelt et al. in which the majority of the evaluated studies consisted of the rehabilitation with 4 implants [5]. The significance of MBL in the present study was interpreted as the more than half of the cases in maxilla was rehabilitated with six implants while it was with 4 implants in mandible mostly. Also, the mean MBL of tilted and axial implants was 0.61 ± 0.63 mm and 0.45 ± 0.41 mm respectively at 1 year. In the second year, MBL of tilted and axial implants was 0.90 ± 0.75 mm and 0.61 ± 0.57 mm respectively. By means of angulation, tilted implants tended to be associated with a greater bone loss but this was not significant at one (*p* = 0.072) and second (*p* = 0.181) years. The mean MBL changes (maxilla as well as mandible) of the present study are consistent with the literature in terms of angulation in 2 years’ follow-up [3, 20, 23–26].

Immediate loading protocol of the dental implants for the rehabilitation of edentulous jaws has been proven with similar survival and success rates with early and conventional loading. By means of MBL, survival and success rates for immediate loading would provide the same outcome as conventional protocols [3, 4, 13, 15]. In the present study, 130 of 179 implants were immediately loaded on the same day and the only implant which failed in the mandible was an immediately loaded implant that resulted in 98.5% CRS in 2 years. At 1 and 2 years, the mean MBL was recorded as 0.53 ± 0.53 mm and 0.67 ± 0.56 mm respectively which was not significantly different from conventional loaded implants in both 1 (*p* = 0.522) and 2 (*p* = 0.099) years and was in accordance with the literature [3, 4, 13, 14].

No consensus has been reached on the most advantageous number of implants to be used to support a fixed dental prosthesis [27]. Malò and colleagues presented the first data following the rehabilitation with four immediately loaded implants in 2005 [28]. The successful results of implant and prosthesis survival/success rates and marginal bone-level changes of this concept were reported in literature. Some researchers reported that six implants could be considered a predictable and cost- and time-effective option for the immediate restoration of the edentulous maxilla [29–31]. Tallarico et al. reported similar MBL and CSR in the maxilla which was rehabilitated with 4 or 6 implants in their 5 years’ follow-up study [32]. Along with all these recent articles, in 1995, P.-I. Brånemark et al. reported the same survival rates with 4 and 6 six implants in edentulous patients while 4 implant scenarios had higher complications [33]. In the present study, patients were mostly rehabilitated with 4 implants in the mandible. In the maxilla, there was an even distribution with 4 and 6 implant-supported solutions. The mean MBL of implants in which the rehabilitation was done with 4 implants was 0.63 ± 0.59 mm (*n* = 80 [24 maxilla/56 mandible]) while it was 0.35 ± 0.33 mm (*n* = 54 [42 maxilla/12 mandible]) in 6 implant-supported prosthesis at 1 year. Even though cumulative survival and success rates were not affected from the variability of the number of implants, MBL in four implant-supported rehabilitations was greater in 1 year (*p* = 0.0060). This result is in accordance with Branemark et al. and Tallarico et al. [32, 33].

The smallest implant diameter for full-arch rehabilitations is 3.3 mm in the literature [19, 32, 34]. In the study of Tallarico et al., four 3.3-mm-diameter implants of total 200 implants were included while Malo et al. placed NDIs only in the anterior region of the maxilla. However, both studies did not mention about the MBL and CSRs of 3.3-mm implants specifically. Piano et al. evaluated twenty-one patients with a total of immediately loaded 84 (74 of 4.1 mm/10 of 3.3 mm) implants (Straumann Bone Level SLActive implants) with the 2-year period. Implant and prosthetic survival rates of 100% were achieved. The mean MBL of 0.34 mm ± 0.45 mm in 2 years was reported. Also, similar marginal bone loss of NDIs and regular-diameter implants was reported (*p* = 0.67) [19].The present study was conducted with the combination of Roxolid Bone Level Tapered NDIs (3.3 mm) and 4.1-mm and 4.8-mm implants. The NDIs (3.3 mm) achieved 0.63 mm (*n* = 58/Sd 0.44 mm) MBL at 1-year data and such result was not significantly different from 4.1 (0.46 mm MBL) and 4.8 (0.32 mm MBL) mm diameter implants. The MBL of NDIs was 1.02 ± 0.74 mm in the second year. The difference between narrow and regular diameter (4.1 mm) was significant in the second year which was clinically acceptable.

In the present study, one implant was lost in the mandible in the provisional prosthesis period which resulted in implant survival as 99.4% for all implants and 98.5% for NDIs at 2 years’ follow-up. According to the implant CSR, many papers in the literature reported clinically acceptable CSRs for tilted and axial implants either in the mandible or the maxilla in the 1- to 5-year period [3, 4, 6, 10, 20, 21, 23–25, 28, 29, 34–38]. Moreover, Patzelt et al. (1 year: 98.6 ± 1.3%, 97.5 ± 1.2% for the maxilla and 99.3 ± 0.7% for the mandible, 2 years: 99.1 ± 1.1%, 98.2 ± 1.1% for the maxilla; and 99.7 ± 0.6% for the mandible) and Soto-Peñaloza et al. (2 years: 99.8%) reported clinically acceptable CSR values for tilted and axial implants either in the mandible or in the maxilla [5, 39]. The CSR of the implants in the present study are consistent with CSR values of implants in the literature.

Out of one lost implant, another 3 implants (2 in the mandible and one in the maxilla) in 3 patients presented biological complications that showed > 4-mm peri-implant pocket, > 2-mm marginal bone loss (MBL), and bleeding on probing. The success rate of all implants in the present study was 98.3% in 2 years. According to the implant success rate, Butura et al. (3 years: 99.66% for the mandible), Graves et al. (16 months: 97.48% for the maxilla), Galindo and Butura (1 year: 99.86% for the mandible), Malo et al. (5 years: 98.1% and up to 10 years: 94.8% for the mandible), Rosen and Gynther (up to 10 years: 97% for the maxilla), Krekmanov et al. (5 years: 100% in mandibula (tilted and axial implants), 98% for tilted implants and 93% for axial implants in the maxilla), Tallarico et al. (up to 7 years: 98.2%), and Degidi et al. (3 years: 97.8% for axial implants and 99.2% for tilted implants in the maxilla) reported clinically acceptable implant success rate values for tilted and axial implants either in the mandible or in the maxilla [11, 26, 35, 40–44]. The success rate of the implants achieved in the present study is accordance with the literature.

Different materials were used in the studies to fabricate immediate fixed provisional prostheses. Some authors used a full-arch acrylic provisional prosthesis reinforced with a titanium or metal framework or with titanium cylinders, while the other authors used all-acrylic prostheses [22, 26, 31, 45, 46]. In the present study, a number of 29 screw-retained fixed provisional all-acrylic prostheses without metal framework were incorporated on the same day. There are many prosthetic mechanical complications such as the fracture of the provisional acrylic prosthesis [39, 47], loosening of prosthetic components [24, 43], and the detachment of an element of the prosthesis [47–49]. In the present study, the fracture of the provisional prosthesis was recorded in 4 patients (14,3%). The incidence of fracture of the provisional prostheses in the present study was 16,1% (5 prostheses) of the total cases. Tooth detachment of the provisional fixed acrylic prosthesis was recorded in two prostheses in two patients, while the screw loosening occurred only in a provisional prosthesis.

In the current study, titanium or chrome-cobalt alloy and ceramic or composite resin were used as a framework and as a veneering material, respectively. Prosthetic CSR was reported between 98.9 and 100% in the literature up to 10 years in the literature [5, 10, 20, 23, 34, 40, 43, 50]. Prosthetic survival in the present study was 100% in 2 years which was similar to those previously reported studies.

Mutually protected occlusion with anterior guidance was used in cases of opposing natural dentition, or tooth and/or implant-supported fixed partial prosthesis as previously described [44]. In all cases of the present study, all fabricated definitive prostheses opposed natural dentition or fixed prosthesis supported by tooth and/or implant not removable prosthesis.

## Conclusion

Narrow-diameter implants have encouraging results in the literature. The results of the present study show that especially in those cases of reduced ridges, the use of narrow-diameter Ti-Zr implants in fixed full-arch rehabilitations seems to be a successful and predictable treatment approach at least in the 2 years’ period by means of CSR and MBL. In order to achieve a better biomechanics distribution of forces in problematic cases, an increase in the number of implants would be a good solution especially in the maxilla. Longer term randomized controlled trials are needed to support the role of NDIs in full mouth fixed immediate rehabilitation.

## Data Availability

The datasets used and/or analyzed during the current study are available from the corresponding author on reasonable request.

## Abbrevıatıons

Ti-Zr: Titanium–Zirconium
NDI: Narrow-diameter implant
CSR: Cumulative survival rate
MBL: Marginal bone loss
FDP: Fixed dental prosthesis
CBCT: Cone beam computed tomography
A-P: Anterior–posterior
Ncm: Newton centimeter
